# Computational Method for Determining the Excess Chemical Potential Using Liquid–Vapor Phase Coexistence Simulations

**DOI:** 10.1021/acs.jpcb.4c07206

**Published:** 2024-12-20

**Authors:** Andrew M. Fadgen, Nicholas A. Pizzi, Rodney J. Wigent, Preston B. Moore

**Affiliations:** Saint Joseph’s University, 600 S. 43rd Street, Philadelphia, Pennsylvania 19104, United States

## Abstract

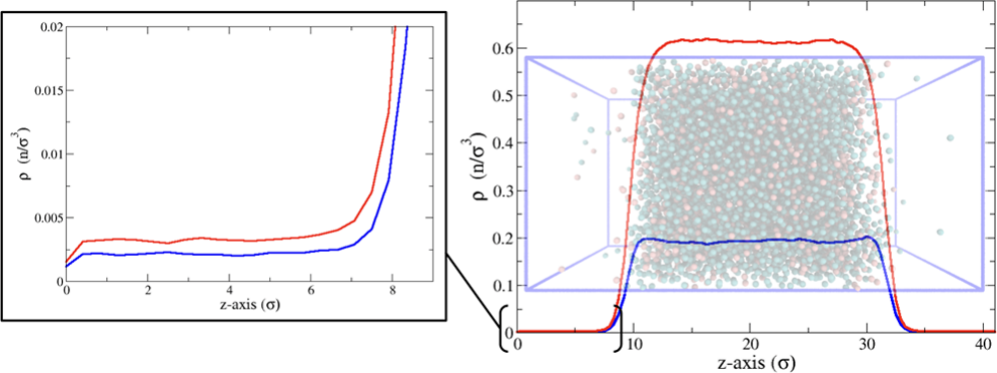

Molecular dynamics simulations are a powerful tool for probing and understanding the theoretical aspects of chemical systems and solutions. Our research introduces a novel method for determining the excess chemical potential of non-ideal solutions by leveraging the equivalence between the chemical potential of the vapor phase and liquid phase. Traditional approaches have relied on bulk simulations and the integration of pair distribution functions (*g*(*r*)), which are computationally intensive to obtain accurate results. In contrast, our method utilizes a liquid–gas system, where determining the vapor pressure allows for a quick and accurate calculation of the excess chemical potential relative to a reference system, e.g., pure solvent. This approach significantly reduces computational effort while maintaining high accuracy and precision. We demonstrate the effectiveness of this method using a simplified Lennard-Jones model, although the method is broadly applicable to a wide range of systems, including those with complex interactions, varying concentrations, and different temperatures. The reduced computational demands and versatility of our approach make it a valuable tool for studying non-ideal solutions, including ionic solutions in molecular simulations.

## Introduction and Background

Chemical potential is fundamental to understanding chemical systems and is essential for analyzing and predicting the behavior of molecular systems under various conditions.^1^ Defined by the fundamental equation of thermodynamics, it represents the change in free energy with respect to the number of atoms of a species .^1^ Chemical potential is crucial for understanding how thermodynamic variables change with chemical composition, making it a key concept in molecular systems. Despite its recognized importance, computing the chemical potential from atomistic simulations remains a challenging task and has not yet become routine.^2^

Although advancements in methods for calculating the chemical potential have been made, each existing computational method offers distinct limitations when being used to examine dense non-ideal solutions. The Widom insertion method, while efficient for dilute systems, is the least suitable for dense, non-ideal liquids due to the low acceptance probability of particle insertions.^3^ Similarly, free energy perturbation struggles with poor convergence when the energy states differ significantly, making it impractical to capture the complexity of large non-ideal systems.^4^ Techniques such as the Grand Canonical Monte Carlo method improve upon these limitations by directly estimating chemical potential through particle fluctuations, but convergence in dense systems remains a challenge.^5^ Methods like adaptive biasing force and umbrella sampling excel at overcoming energy barriers and exploring poorly sampled regions, but their effectiveness comes at the cost of extensive parametrization and computational expense.^6,7^ Alchemical free-energy methods and histogram re-weighting provide detailed free-energy data, but demand extensive sampling and are sensitive to finite-size effects.^8,9^ Thermodynamic integration, while versatile and accurate, requires substantial computational resources, making it inefficient for routine analysis of non-ideal liquids.^10^ Amid these methods, Kirkwood–Buff integration (KBI) stands out as the optimal approach for examining large dense non-ideal liquid solutions at equilibrium.^11−13^ By linking molecular structure directly to thermodynamic properties through integrals of pair correlation functions, KBI captures the intricacies of solute–solvent interactions directly. While it demands significant sampling and careful handling of finite-size effects, its balance of computational feasibility and insightful results makes it a good benchmark for studying non-ideal liquid solutions. Reliable convergence of KBI for each of the solutions examined was achieved through the Lennard-Jones (LJ) cutoff distance σ = 7 and additional *g*(*r*) corrections.^14,15^

Simulations of liquid–vapor systems have been conducted for decades; however, these simulations are traditionally used to understand surface tension and the interfacial region between bulk liquid and vapor phases.^16,17^ When chemical potentials are considered, the methods outlined above calculate this value by utilizing the correspondence of chemical potential between the bulk liquid and vapor components.^18^ By utilizing this correspondence, we show how chemical potential can be calculated efficiently over the entire range of mole fractions. We show that this liquid–vapor method is an effective and efficient way of calculating the excess chemical potential.

The chemical potential of a system can be expressed as μ_i_ = μ_i_^*^ + *RT* ln (*a*_i_), where μ_i_^*^ is the reference state of species *i*, *R* is the gas constant, *T* is the temperature, and *a*_i_ is the activity. The activity *a*_i_ is defined as *a*_i_ = γ_i_*x*_i_, where *x*_i_ is the mole fraction, and γ_i_ is the activity coefficient. Chemical activity (*a*_A_) measures the effective concentration of a species in a mixture, accounting for non-ideal behavior due to molecular interactions. In liquids and gases, the chemical potential can be described as μ_i_ = μ_i_^*^ + *RT* ln (*x*_i_) + *RT* ln (γ_i_). Under ideal conditions, γ_i_ = 1, making the last term zero. To express deviations from ideality, the chemical potential is often represented as the sum of an ideal and an excess term: μ_i_ = μ_i_^id^ + μ_i_^ex^, where μ_i_^ex^ = *RT* ln (γ_i_).

Experimentally, the activity of a solute can be measured by its vapor pressure, where *a*_i_ = *P*_i_/*P*_i_^*^, with *P*_i_ being the vapor pressure and *P*_i_^*^ being the vapor pressure of the pure substance. The activity coefficient γ_i_ is then given by γ_i_ = *a*_i_/*x*_i_, quantifying the deviation from ideality for the species. Since the chemical potentials of the gas phase and liquid phase are identical at equilibrium (μ_gas_ = μ_liquid_), knowing the gas-phase chemical potential allows for the determination of the liquid-phase chemical potential.

Inspired by experimental methods in which the activity of liquids is measured via vapor pressure, we propose a method for computing activities and chemical potentials by leveraging the relationship between the vapor phase and the liquid phase. This approach is similar to previous techniques used for measuring molecular volume changes.^19^ Here, we demonstrate the effectiveness of calculating the excess chemical potential of a solution using a simplified model such as the LJ potential. While our study employs a simplified model as a proof of concept, this method is broadly applicable to a variety of systems and can accommodate different concentrations, complex interactions, and varying temperatures. Compared with traditional methods that rely on KBIs in simulations, our approach requires reduced computational effort. KBIs have also been theoretically derived for multiple component systems, including fourth and higher component mixtures and more complex fluids, such as water.^20,21^ Our liquid–vapor method is also applicable to multiple-component systems and water. We show that the liquid–vapor method yields accurate and precise results for this system, and we believe that it will achieve similar accuracy in more complex simulations.

All simulations were performed using LAMMPS.^22^ The simulations employed reduced LJ units with σ = 1 and a LJ cutoff of 7σ. A total of 8000 LJ particles were simulated under periodic boundary conditions, with a reduced temperature of 0.75. The interactions between A–A and B–B particles were set with σ = 1 and ε = 1, while the A–B interactions were varied with σ = 1 and ε = 0.9, 1.0, and 1.1. The mole fraction of species A (*X*_A_) was varied from 0 to 1, with increments of *X*_A_ at 0, 0.02, 0.04, 0.06, 0.08, 0.10, 0.12, 0.14, 0.16, 0.18, 0.20, 0.25, 0.35, and 0.50. Given that A and B have identical interactions (σ = 1, ε = 1), the labels can be switched to obtain additional mole fractions of 0.65, 0.75, 0.80, 0.82, 0.84, 0.86, 0.88, 0.90, 0.92, 0.94, 0.96, 0.98, and 1.

**Figure 1:**
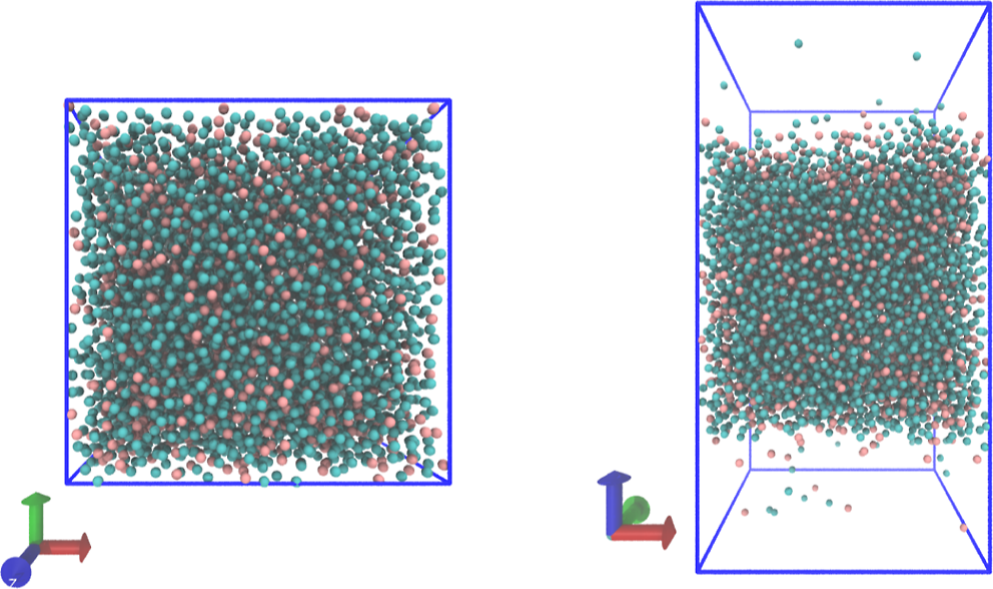
“Bulk” configuration of a 25% A (red) and 75% B (blue) solution (left) and “slab” configuration of a 25% A (red) and 75% B (blue) solution (right).

We initially simulated a bulk liquid solution of LJ particles in the NPT ensemble for each mole fraction of species A. These bulk simulations were used to generate the radial distribution functions (*g*(*r*)) and KBIs for each species pair. Using an equilibrated bulk configuration, slab configurations were created by expanding the *z*-axis in each direction by 10 units (see Figure 1, which depicts both bulk and slab configurations). In both bulk and slab configurations, we performed 20,000 equilibration steps. After equilibrating the system, as assessed by the stabilization of pressure and volume, we ran simulations for 1 million steps, storing configurations every 1000 steps for analysis.

**Figure 2:**
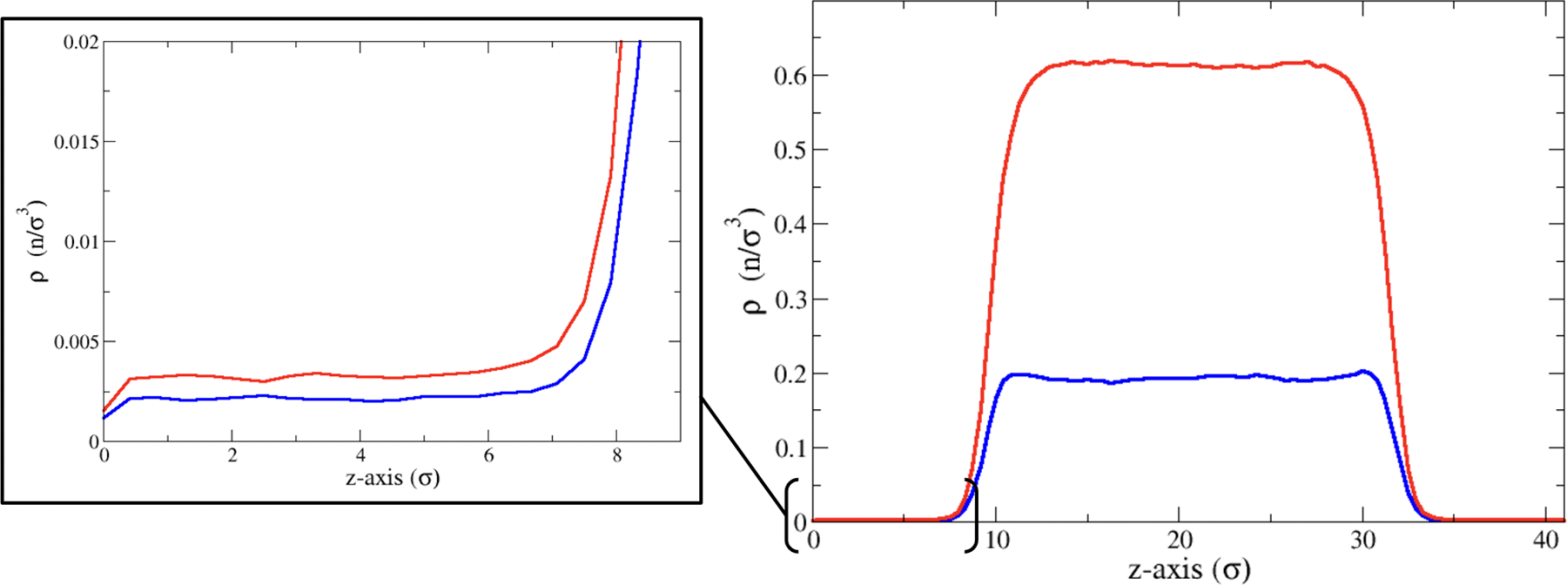
Density of 25% A (red) and 75% B (blue) solution in the slab conformation vs the *z*-axis.

As stated previously, activity is defined as the ratio of the pressure of a system to the pressure of a pure substance. However, we can approximate the vapor phase as nearly ideal (e.g., *P* ≈ (*n*/*V*)*RT*) with minimal interactions, allowing the chemical activity to be related to the vapor density (*n*/*V*).^23^ By this approximation, the activity can be expressed as the ratio of the vapor density of the component in the mixture to the vapor density of the pure component at the same temperature and pressure. In our simulations, we determine the density profiles of species A and B along the *z*-axis in the slab configuration. As shown in Figure 2, the vapor density is significantly lower than the liquid density, but the vapor density is still readily measurable from the simulations.

## Derivative of Excess Chemical Potential in Terms of the Natural Log of the Activity Coefficient

The chemical potential of A in an ideal solution is μ_A_^id^ = μ_A_^°^ + *RT* (ln (*X*_A_)), while for a non-ideal solution it is μ_A_ = μ*A*° + *RT* (ln (γ_A_*X*_A_)). Solving for the excess chemical potential of A in terms of activity coefficient yields μ_A_^ex^ = μ_A_ – μ_A_^id^ = *RT* (ln (γ_A_)), and taking the derivative with respect to the mole fraction gives . This derivation will be used to compare the KBI and liquid–vapor methods.

**Figure 3:**
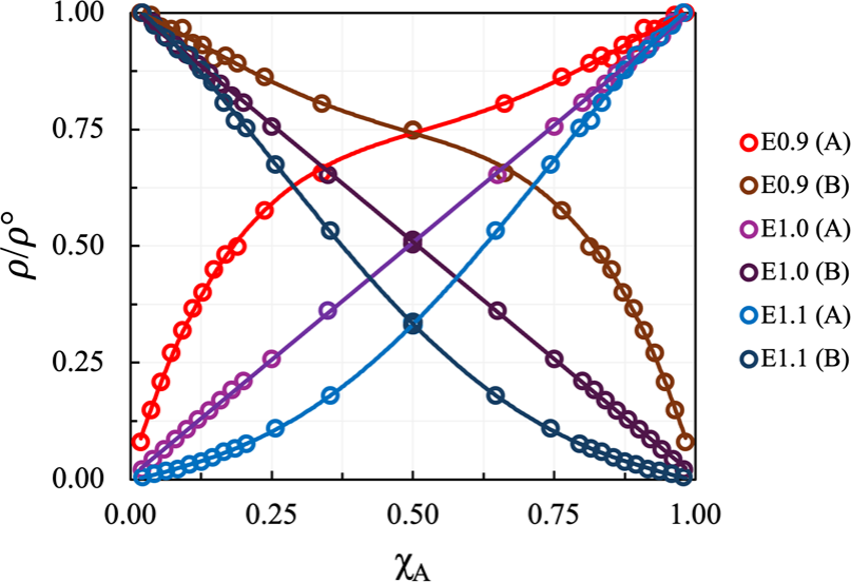
Quotient of the average vapor density of A and B divided by the average vapor density of A and B in a pure solution for *E* = 0.9, 1.0, and 1.1 vs *X*_A_.

In Figure 3, the activity (ρ/ρ°) is plotted as a function of the mole fraction of the liquid. It is important to note that the mole fraction of the vapor differs from that of the liquid. In this closed system, containing a total of 8000 particles, changes in the number of particles in the vapor phase directly influence the number of particles in the liquid phase. However, the ratios of these changes are not identical between the two phases. We have used the liquid phase mole fraction as the reference since it closely approximates the total mole fraction.

## Solving the Kirkwood–Buff Solution in Terms of the Derivative of the Natural Log of the Activity Coefficient with Respect to *X*_A_

The seminal work of Kirkwood and Buff,^24,25^ along with contributions from others, established a relationship between structure factors and thermodynamic quantities.^26,27^ Ben–Naim demonstrated that the Kirkwood–Buff theory could be inverted, allowing thermodynamic data to be used to obtain the values of the KBIs.^28^

Chemical potential can be related to the KBI as
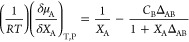
1where *X*_A_ is the mole fraction, *C*_B_ is the number density of *B*, and Δ_AB_ is the difference between the pure and paired KBI (Δ_AB_ = *G*_AA_ + *G*_BB_ – 2*G*_AB_). As described in the literature, when Δ_AB_ = 0, the solution behaves ideally (often referred to as symmetric ideal), even though the conditions for ideal behavior are not met.^28^ For example, the chemical potential can be described as μ_id,A_ = μ_A_° + *RT* ln (*X*_A_) despite the presence of strong interactions between the chemical species.

The derivative of the natural log of the activity coefficient with respect to *X*_A_ can be related to by
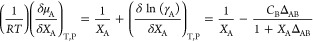
2which leads to
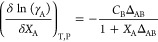
3

**Figure 4:**
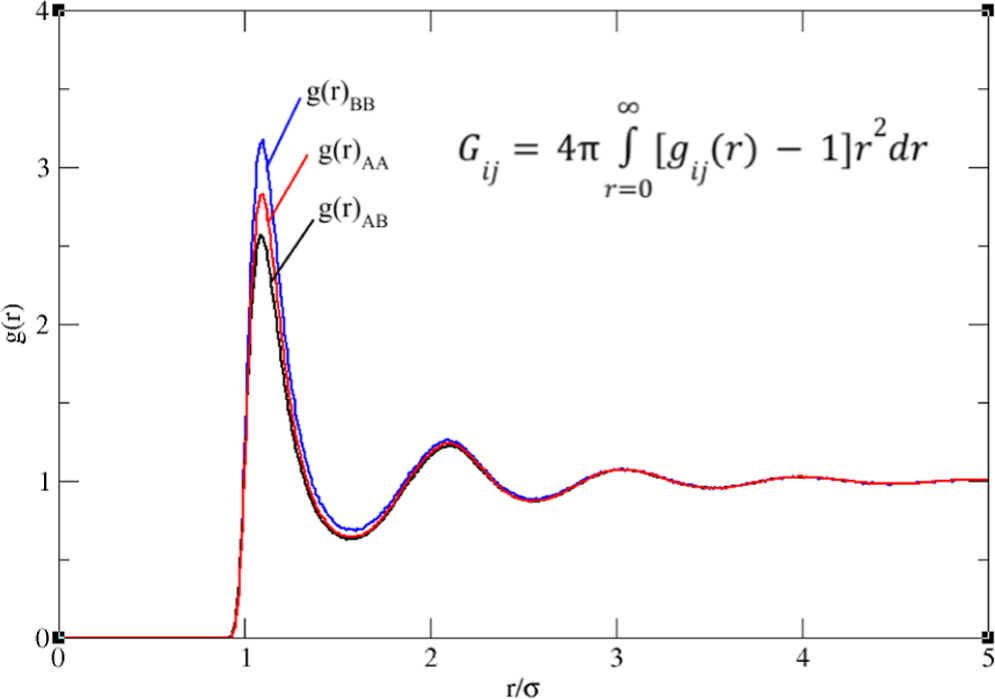
Radial distribution function of pairs *G*_AA_, *G*_AB_, and *G*_BB_ (25% A, 75% B, *T* = 0.75, ε = 0.9), the KBIs are related to the difference in these curves, which are prone to sampling error, finite size effects, and numerical noise, especially at large *r*.

The pair distribution functions (*g*(*r*)) from the bulk simulations are shown in Figure 4. The KBIs are obtained by integrating these functions, and the activity coefficient is calculated from the differences in these integrals. Although the KBI theory is very powerful, there are several potential sources of error when computing the values from the simulation data. For instance, noise in the *g*(*r*) becomes amplified as *r* increases due to the volume element of the integral (*r*^2^ d*r*). Additionally, finite size effects and corrections depending on whether the system is open or closed can further complicate the calculations.^29,30^ These challenges make it difficult to obtain accurate KBI values, and the error is compounded when calculating the activity coefficient from simulations by this method, as the necessary subtractions may fall within the margin of error. However, due to the simplicity of our system, we achieved convergence (Figure 4) by using large cutoffs for the potentials and careful implementation of algorithms to reduce roundoff errors.

## Comparing the Liquid–Vapor Method to the Kirkwood–Buff Method

As shown previously, by taking the derivative of the excess chemical potential of species A with respect to its mole fraction, one can equate the terms used in the KBI method to the activity coefficient obtained from the liquid–vapor method:
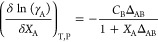
4

**Figure 5:**
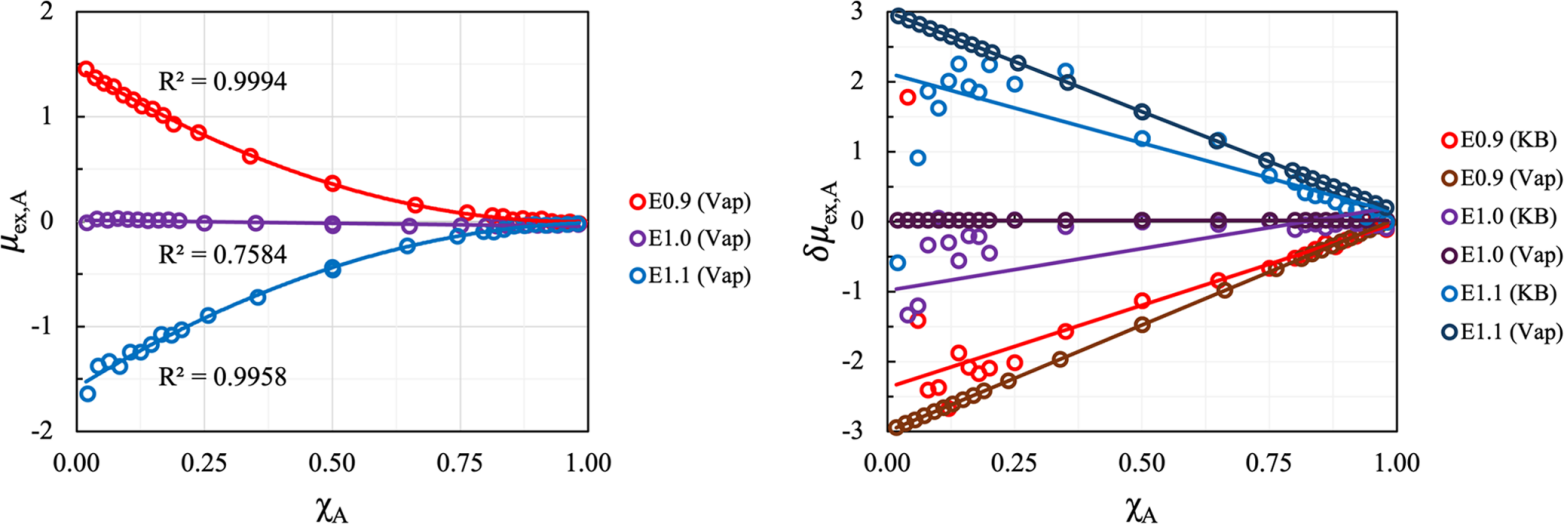
Excess chemical potential of A for the liquid–vapor method vs *X*_A_ (left) and the derivative of excess chemical potential of A for the KBI and liquid–vapor method vs *X*_A_ (right).

Using data from trials where ε = 0.9, 1.0, and 1.1 from both KBI method and the liquid–vapor method, we can establish a system of equivalent equations to compare the two approaches. Figure 5 illustrates the derivative of the excess chemical potential of species A with respect to *X*_A_, calculated using both KBIs and vapor density data. We find that the methods are in quantitative agreement with the calculated derivatives of the excess chemical potential falling within the error margins of both calculations.

Interestingly, we observe a linear relationship between the derivative of the excess chemical potential and the mole fraction, which aligns with the predictions of the Margules equations. The Margules model is often used to describe the behavior of non-ideal solutions, where the excess Gibbs free energy for a binary mixture can be written as *G*^ex^ = ξ *x*_A_*x*_B_, where ξ is the Margules interaction parameter, and *x*_A_ and *x*_B_ are the mole fractions of components A and B, respectively. Thus, one obtains that . This relationship suggests that for our simple LJ systems, the Margules model accurately describes the non-ideal behavior. In this context, the slope of the linear relationship between the excess chemical potential corresponds to ξ, while the y-intercept represents 2ξ.

## Finite-Size Effects

Finite-size effects are inherent in molecular dynamics simulations due to the limited number of particles and the finite volume of the simulation box.^31,32^ These effects can significantly impact various properties, particularly those related to long-range interactions and phase behavior. For example, finite-size effects can lead to deviations in calculated properties, such as pressure, density, and structure factors, especially in systems where long-range forces play a crucial role. To mitigate these effects and improve the accuracy of the simulation results, several techniques can be employed. Increasing the system size can help to reduce the relative impact of finite-size effects by providing a larger volume that better approximates the behavior of an infinite system. Additionally, using advanced algorithms and enhanced sampling methods can improve the efficiency and accuracy of simulations by allowing for better exploration of phase space and reducing statistical errors.^12,29,30^

In the liquid–vapor method, finite size effects do not significantly impact the average vapor density value. Trials conducted with varying numbers of atoms, but consistent mole fractions of species A and B, reliably show that the average vapor density remains independent of the simulation size (see Table 1). While the standard deviation of vapor densities decreases, indicating improved internal consistency within individual trials, the average vapor density values themselves remain unchanged. These findings demonstrate that finite-size effects do not significantly influence the average vapor densities obtained through the liquid–vapor method.

**Table 1:** Vapor Density for Species *B* (μ_ex,B_) for 4K, 8K, and 32K Atoms and ε = 0.9, 1.0, and 1.1 in a Solution of 20% A and 80% B

epsilon (E)	4K atoms	8K atoms	32K atoms
0.9	0.00347 ± 0.00011	0.003416 ± 0.000069	0.003405 ± 0.000024
1.0	0.00311 ± 0.00015	0.003033 ± 0.000050	0.003116 ± 0.000031
1.1	0.00285 ± 0.00010	0.002817 ± 0.000043	0.002847 ± 0.000026

## Computational Efficiency

While a similar number of configurations were used for calculating both slab and bulk systems, the liquid–vapor method demonstrates significantly higher efficiency compared to the Kirkwood–Buff method during data analysis. The Kirkwood–Buff method’s radial distribution functions are often affected by substantial statistical noise, requiring extensive, trial-specific preparation techniques for accurate analysis.^2,12^ In contrast, the liquid–vapor method can achieve a sufficient data set without extensive simulation or trial-specific preparation and can be analyzed using a broadly applicable technique. The primary limitation of the liquid–vapor method is the computational power of the device used. Although equilibrium was reached quickly within the LJ systems observed, the time taken to reach equilibrium in more complex solutions is a potential limitation of the liquid–vapor method. Anecdotally, we observe that as long as there are vapor-phase species at the interface, chemical equilibrium is reached relatively quickly.

## Conclusions

In conclusion, our study highlights the advantages of the liquid–vapor method over traditional Kirkwood–Buff methods for calculating excess chemical potentials and analyzing non-ideal solutions. The liquid–vapor method achieves results that are in quantitative agreement with those obtained by using the KBI method, demonstrating that it can produce accurate and reliable data while significantly improving efficiency and less computational complexity. In contrast, the Kirkwood–Buff approach, while powerful, is hampered by significant statistical noise and the need for extensive preparation and analysis. The liquid–vapor method’s efficiency and reduced sensitivity to finite-size effects make it valuable for simulations across a range of conditions. Overall, this study demonstrates that the liquid–vapor method provides a practical and accurate alternative for studying the excess chemical potential in molecular simulations, offering clear advantages in both efficiency and reliability.
